# Synthesis of water-dispersible, plate-like perovskites and their core–shell nanocrystals†

**DOI:** 10.1039/d0ra00657b

**Published:** 2020-02-05

**Authors:** Muneharu Minakawa, Yoshiro Imura, Takeshi Kawai

**Affiliations:** Department of Industrial Chemistry, Tokyo University of Science 1-3 Kagurazaka, Shinjuku-ku Tokyo 162-8601 Japan kawai@ci.kagu.tus.ac.jp

## Abstract

Shape-controlled halide perovskite nanocrystals are attractive as an emerging functional material; however, these nanocrystals are prepared using organic solvents containing alkylamines and there are few reports on the synthesis of water-dispersible halide perovskite nanocrystals. We report a simple method to prepare water-dispersible, plate-like perovskite nanocrystals by mixing a long-chain amidoamine derivative (C18AA) and potassium tetrachloropalladate (K_2_PdCl_4_) in water. The obtained nanocrystals have a 2D layered perovskite structure represented by the chemical formula (C18AAH_2_)PdCl_4_. Furthermore, because seed-mediated growth is useful for preparing shape-controlled nanocrystals, such as rods, plates, wires and cubes, we used the water-dispersible (C18AAH_2_)PdCl_4_ nanocrystals as seeds to grow (C18AAH_2_)PdCl_4_@Pt core–shell nanocrystals. The core–shell nanocrystals have rough surfaces due to the deposition of Pt on the (C18AAH_2_)PdCl_4_ seeds. In addition, plate-like (C18AAH_2_)PdCl_4_@Au core–shell nanocrystals were easily obtained using this seed-mediated growth method.

## Abstract 2

Water-dispersible, plate-like perovskite nanocrystals were prepared using a long-chain amidoamine derivative (C18AA) and perovskite@Pt or Au core–shell nanocrystals were synthesized using the plate-like perovskite nanocrystals as seeds.

## Graphical abstract

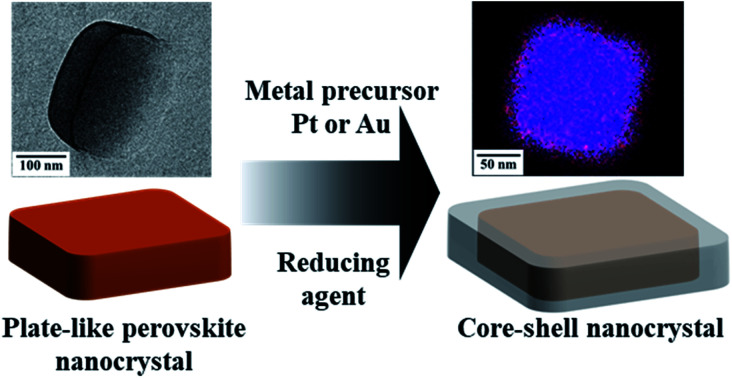

## 1. Introduction

Noble metal nanocrystals have attractive electrical, magnetic and optical properties that have led them to be used in a wide range of practical applications, including electronics, biotechnology and catalysis.^1–5^ Because the physical and chemical properties of these nanocrystals are strongly influenced by their shapes, controlling their sizes and shapes is naturally recognized as an important research area,^6–8^ and many methods to synthesize shape-controlled nanocrystals, such as rods,^9–11^ plates,^12,13^ wires,^14,15^ cubes^16,17^ and flowers,^18,19^ have been developed in the past several decades. Furthermore, nanocrystal properties are also significantly influenced by coating a sheath of another material because the shell material imparts additional functionality to the core nanocrystal. Accordingly, applying the core–shell technique to shape-controlled materials can be used to create unique nanocrystals with multifunctional capabilities derived from both the core and shell materials. Recently, core–shell nanocrystals with shape-controlled core materials, which form a new class of anisotropic nanostructure, have been extensively prepared using wet chemical methods in water.^20–24^

Recently, organic–inorganic halide perovskite nanocrystals with low dimensionalities, which are emergent functional materials with highly efficient electronic and optoelectronic properties, have been studied extensively.^25–28^ Here, the structure of the organic–inorganic halide perovskite is denoted as (RNH_3_)_*m*_MX_*n*_, where RNH_3_ is an amine derivative, M is a metal and X is a halide. The electrical and optical properties depend on the morphology, size and compositions of the nanocrystals;^29,30^ controlling the morphology is therefore crucial for the crystal engineering of (RNH_3_)_*m*_MX_*n*_. According to previous reports,^31–33^ anisotropically shaped perovskites, such as rods, plates and wires, have been successfully prepared using organic solvents containing water-insoluble amine derivatives (RNH_2_); consequently the as-prepared perovskites do not disperse in water, which is an environmentally friendly solvent.

So far, few syntheses of water-dispersible perovskites have been reported due to the limited number of available water-soluble dispersants that can stabilise (RNH_3_)_*m*_MX_*n*_ structures.^34,35^ Furthermore, water-dispersibility is absolutely essential for preparing core–shell structures because the shell is generally produced in the water phase. Actually, an experimental attempt to prepare a metal-shell covering on water-insoluble (CH_3_NH_3_)_2_PdCl_4_ nanocrystals failed (Fig. S1†). As a natural consequence, fabricating water-dispersible core–shell nanocrystals with anisotropic perovskite cores is a challenging issue.

**Fig. 1:**
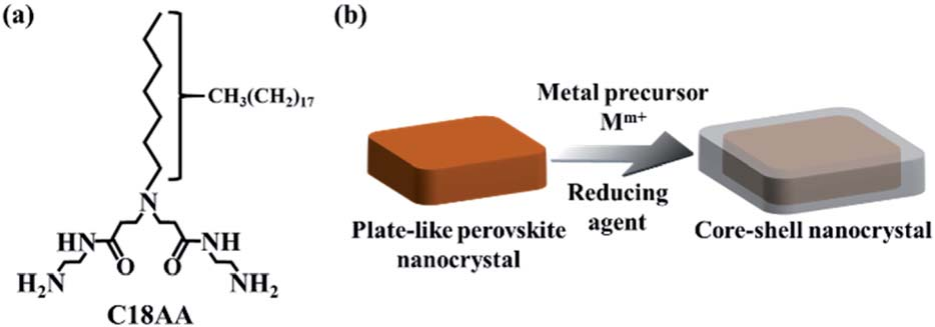
(a) Molecular structure of C18AA. (b) Schematic illustration of water-dispersible, plate-like perovskite@M core–shell nanocrystals.

We previously demonstrated that a water-soluble, long-chain amidoamine derivative (C18AA, Fig. 1a) is a highly effective and functional noble metal nanocrystal dispersant because it is capable of dispersing such nanocrystals in both aqueous and organic phases.^36^ C18AA can also reversibly transfer the nanocrystals between the two phases by tuning the pH.^37^ Using water-soluble C18AA instead of water-insoluble alkylamines enables the preparation of water-dispersible perovskite nanocrystals. In this study, we demonstrate that water-dispersible, plate-like perovskite nanocrystals can be prepared by simply mixing C18AA and potassium tetrachloropalladate (K_2_PdCl_4_) in water. Further, we also show that the plate-like nanocrystals can be used as seeds to grow core–shell nanocrystals (Fig. 1b).

## 2. Experimental

### 2.1. Materials

Potassium tetrachloropalladate (K_2_PdCl_4_) and potassium tetrachloroplatinate (K_2_PtCl_4_) were purchased from Kanto Chemical. Hydrogen tetrachloroaurate tetrahydrate (HAuCl_4_·4H_2_O) was purchased from Nacalai Tesque. Ascorbic acid (AscA), and sodium borohydride (NaBH_4_) were obtained from Tokyo Chemical Industry. C18AA and derivatives with different chain lengths (C16AA and C14AA) were synthesised using a previously reported method.^38^

### 2.2. Preparation of perovskite nanocrystals

Water-dispersible (C18AAH_2_)PdCl_4_ nanocrystals were prepared by mixing a 20 mM aqueous solution of C18AA (500 μL) with a 20 mM aqueous solution of K_2_PdCl_4_ (500 μL) in a 2 mL glass bottle. The mixture was placed in a water bath at 80 °C for 1 min to completely dissolve the (C18AAH_2_)PdCl_4_ nanocrystals. The water-insoluble (CH_3_NH_3_)_2_PdCl_4_ perovskite was prepared by a previously reported method.^39^

### 2.3. Preparation of perovskite@Pt core–shell nanocrystals

A 200 mM aqueous solution of K_2_PtCl_4_ (50 μL) and a 600 mM aqueous solution of AscA (50 μL) were added to a 10 mM aqueous dispersion of (C18AAH_2_)PdCl_4_ nanocrystals (1000 μL). The mixture was left at room temperature for one day. The molar ratio of AscA : K_2_PtCl_4_ was 3 : 1. The same procedure was applied to the (CH_3_NH_3_)_2_PdCl_4_ perovskite instead of the (C18AAH_2_)PdCl_4_ nanocrystals.

#### 2.3.1. Effect of the K_2_PtCl_4_ molar concentration

To determine the effect of the K_2_PtCl_4_ molar concentration on the structure of the perovskite@Pt core–shell nanocrystals, a 200 mM K_2_PtCl_4_ aqueous solution (5, 12.5 or 25 μL) was added to a 10 mM aqueous dispersions of perovskite nanocrystals (1000 μL). A 600 mM AscA aqueous solution (50 μL) was also added to the mixture, which was then left at room temperature for one day. The molar ratios of AscA : K_2_PtCl_4_ were 6 : 1, 12 : 1 and 30 : 1, respectively.

#### 2.3.2. Effect of the AscA molar concentration

To determine the effect of the AscA molar concentration on the perovskite@Pt structure, the preparation of core–shell nanocrystals described in 2.3 (above) was carried out using a 600 mM AscA aqueous solution (100, 200 and 500 μL). The molar ratios of AscA : K_2_PtCl_4_ were 6 : 1, 12 : 1 and 30 : 1, respectively.

#### 2.3.3. Effect of the use of NaBH_4_ as a reducing agent

To determine the effect of using NaBH_4_ as the reducing agent instead of AscA on the perovskite@Pt structure, the preparation of core–shell nanocrystals described in 2.3.1 (above) was carried out using a 600 mM NaBH_4_ aqueous solution (50, 100, 200 and 500 μL). The molar ratios of NaBH_4_ : K_2_PtCl_4_ were 3 : 1, 6 : 1, 12 : 1 and 30 : 1, respectively.

### 2.4. Preparation of perovskite@Au core–shell nanocrystals

A 240 mM aqueous solution of HAuCl_4_ (4.2 μL) and a 100 mM aqueous solution of AscA (100 μL) were added to a 10 mM aqueous dispersion of (C18AAH_2_)PdCl_4_ nanocrystals (1000 μL). The mixture was left at room temperature for one day. The molar ratio of AscA : HAuCl_4_ was 10 : 1. The same procedure was applied to the (CH_3_NH_3_)_2_PdCl_4_ perovskite instead of the (C18AAH_2_)PdCl_4_ nanocrystals.

### 2.5. Characterisation

Transmission electron microscopy (TEM) was carried out using a JEOL JEM-1011 instrument operating at 100 kV. Scanning TEM mapping was performed using a JEOL 2100 instrument equipped with an energy-dispersive X-ray spectrometer (EDS) operated at 200 kV. Scanning electron microscopy (SEM) was performed with a Hitachi S4800 microscope operating at 20 kV. X-ray diffraction (XRD) patterns were recorded on a Rigaku Ultima IV diffractometer with Cu Kα radiation (*λ* = 0.15405 nm). X-ray photoelectron spectroscopy (XPS) was performed with a JEOL JPS-9010MC XP spectrometer equipped with a Mg Kα X-ray source. CHN elemental analysis was conducted with a PerkinElmer 2400 II CHNS/O. Inductively coupled plasma-mass spectrometry (ICP-MS) was performed with an Agilent 7500 instrument from Agilent Technologies. Thermogravimetric (TG) analysis was conducted using a TG/DTA 6200 instrument.

## 3. Results and discussion

### 3.1. Preparation of water-dispersible perovskite nanocrystals

**Fig. 2:**
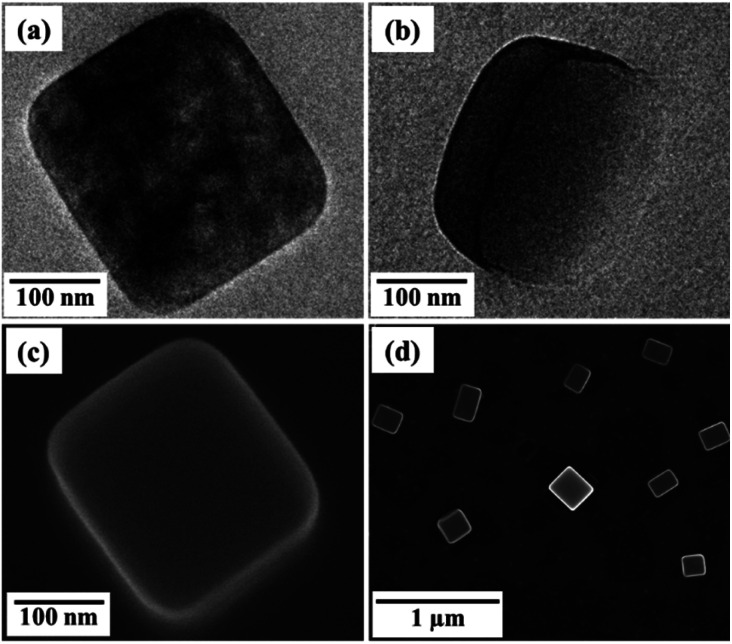
(a and b) TEM images and (c and d) SEM images of water-dispersible, plate-like perovskite nanocrystals.

Mixing C18AA with K_2_PdCl_4_ instantly made an opaque dispersion containing nanocrystals, and the dispersion was stable for at least six months. Fig. 2 shows TEM images of the product obtained using 20 mM solutions of C18AA and K_2_PdCl_4_. These images clearly reveal that the product consists of plate-like nanocrystals with an average size (*s*) of 153 ± 65 nm and a thickness (*t*) of 50 ± 24 nm (Fig. S2†). The sizes and the size distribution of the nanocrystals did not change with C18AA and K_2_PdCl_4_ concentrations as long as the molar ratio of C18AA to K_2_PdCl_4_ was 1. Using molar ratios of 2, 5 or 10 resulted in plate-like nanocrystals similar to the products obtained with a molar ratio of 1, but when the molar ratio was 0.5, irregularly shaped crystals were obtained instead of plate-like ones.

**Fig. 3:**
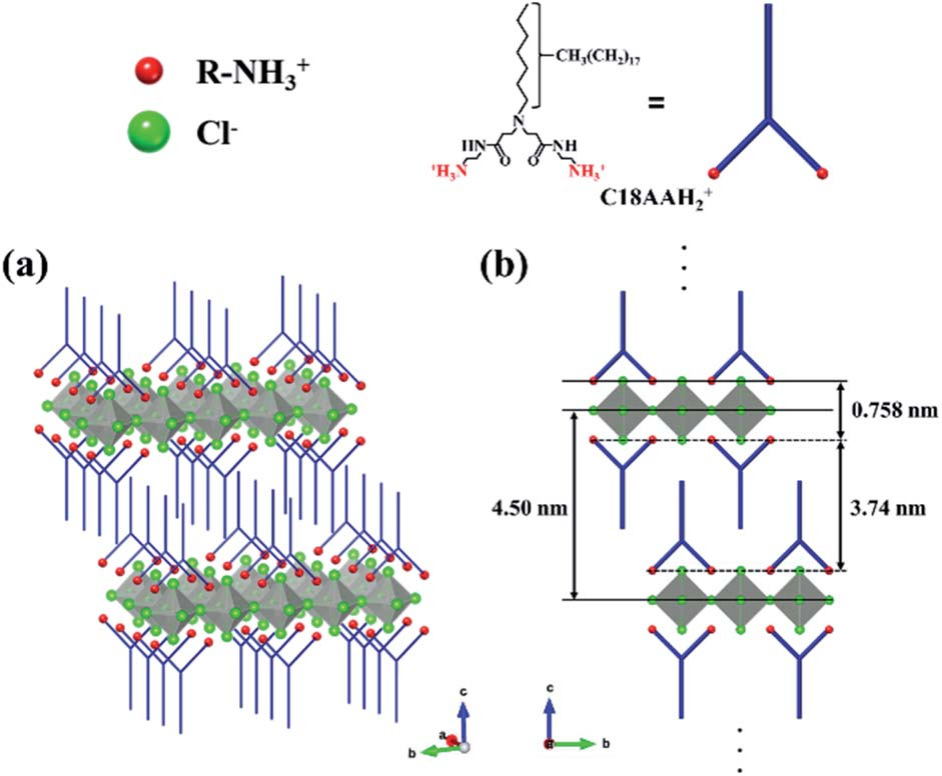
(a) 3D and (b) 2D images of the crystal structure of (C18AAH_2_)PdCl_4_.

**Fig. 4:**
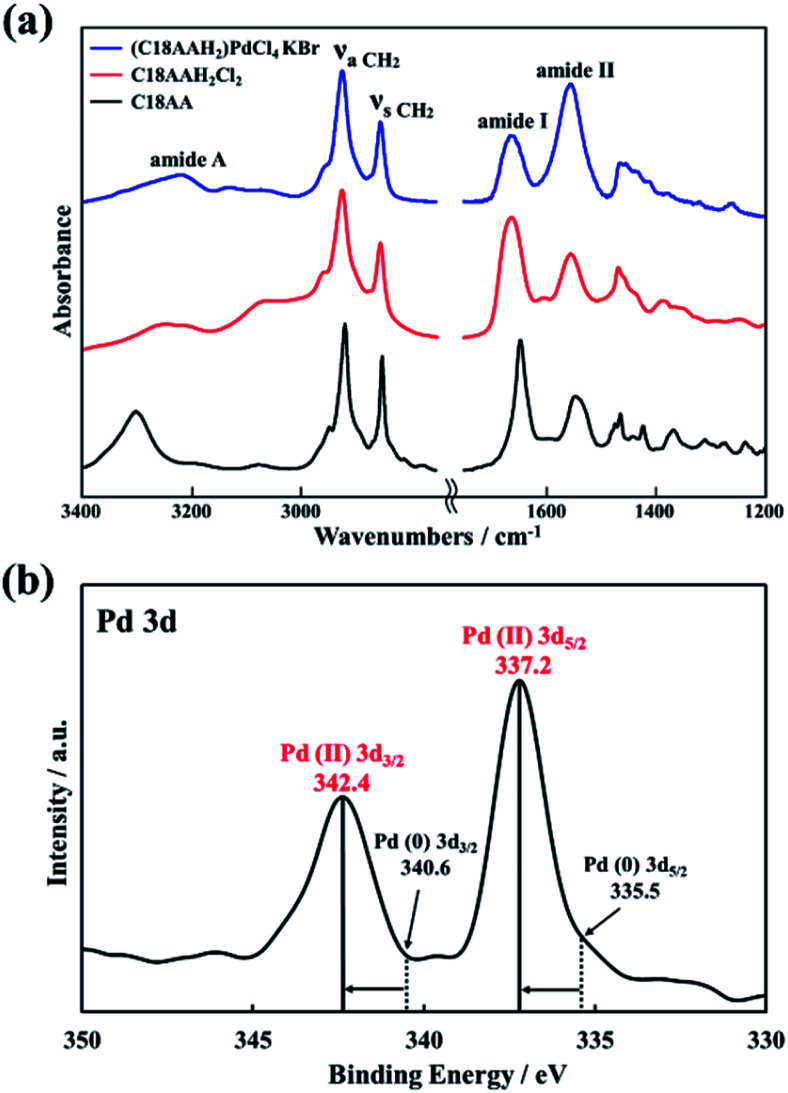
(a) FT-IR and (b) XPS spectrum of the plate-like nanocrystals.

In general, the chemical formula of a two-dimensional (2D) organic–inorganic layered perovskite prepared from a metal halide and an alkylamine is A_2_BX_4_, where A is the ammonium cation of the alkylamine (R–NH_3_^+^), B is the metal ion, and X is the halide. The elemental ratio of the nanocrystals evaluated by CHN elemental analysis and ICP-MS was C/H/N/Pd/Cl = 28.0/60.7/5.02/1/3.92 (Table S1†), which is consistent with the theoretical molar ratio of C18AAH_2_(C_28_H_61_N_5_O_2_) : Pd : Cl = 1 : 1 : 4 and the structure of the (C18AAH_2_)PdCl_4_ 2D layered perovskite (Fig. 3). The molar ratio of C18AAH_2_^2+^ : PdCl_4_^2−^ = 1 : 1 was also in good agreement with the thermogravimetric-analysis data for the nanocrystals (Fig. S3†). Furthermore, the chemical components in (C18AAH_2_)PdCl_4_, namely C18AAH_2_^2+^ (protonated C18AA) and Pd^2+^, were also confirmed by FT-IR spectroscopy and XPS. Fig. 4a shows FT-IR spectra of C18AA, protonated C18AA and the plate-like nanocrystals. Clearly, the spectrum of the nanocrystals is similar to that of the protonated C18AA (Table S2†). This estimation is also in good agreement with the XPS results. Pd 3d peaks are evident in the XPS spectrum of the plate-like nanocrystals at binding energies of 337.2 and 342.4 eV (Fig. 4a), and are assigned to 3d_5/2_ and 3d_3/2_ of Pd(ii), respectively, because Pd(ii) peaks are observed at 337.2 and 342.4 eV in the spectra of layered perovskites,^40^ whereas they are at 335.5 and 340.6 eV for Pd(0).^40^ These result supports that the obtained nanocrystals are perovskites represented by chemical formula of (C18AAH_2_)PdCl_4_.

Typical 2D organic–inorganic perovskites with periodic layered structures show characteristic periodic patterns in their XRD patterns originating from the (00*l*) planes. (CH_3_NH_3_)_2_PdCl_4_ 2D perovskites composed of methyl ammonium cation and palladium chloride, for instant, show periodic peaks of the (00*l*) plans at 9.72°, 19.48° and 29.40°, which correspond to a *d* spacing of 0.90 nm (Fig. S4†).^39^ The present plate-like (C18AAH_2_)PdCl_4_ nanocrystals show diffraction peaks at 1.96° and 3.88° assigned to the (001) and (002) planes (Fig. S5†), although the higher index peaks could not be detected in the XRD spectrum. This result indicates that (C18AAH_2_)PdCl_4_ surely has a periodic layer structure with a *d* spacing of 4.50 nm, although the crystallinity of the layered direction (*c* axis) is not high.

The proposed structure of (C18AAH_2_)PdCl_4_ shown in Fig. 3 was determined based on the fact that the XRD pattern in the small-angle region of (C18AAH_2_)PdCl_4_ showed that its crystal structure is similar to that of (CH_3_NH_3_)_2_PdCl_4_, except for the periodic layer length. In a previous XRD study of (CH_3_NH_3_)_2_PdCl_4_,^39^ the thicknesses of the CH_3_NH_3_^+^ and PdCl_4_^2−^ layers were determined to be 0.151 nm and 0.758 nm, respectively (Fig. S6†). Assuming that the thickness of the PdCl_4_^2−^ layer in (C18AAH_2_)PdCl_4_ is the same as that in (CH_3_NH_3_)_2_PdCl_4_, the C18AA layer can be determined to be 4.50–0.758 = 3.74 nm thick. As C18AA was estimated from the CPK model to have a molecular length of approximately 2.8 nm,^38^ the thickness of C18AA exceeds, but is less than double the molecular length. This implies that the hydrocarbon chains are in an interdigitated state. This proposal is consistent with the fact that the head groups of C18AA are bulkier than the hydrocarbon tail group. The interdigitated structure of the carbon chains in (C18AAH_2_)PdCl_4_ give rise to strong binding interactions between layers, whereas the carbon chains do not adopt an interdigitated structure in 2D organic–inorganic perovskites prepared with single-chain amine derivatives, but rather adopt a face-to-face configuration of the terminal methyl groups.

Similar plate-like nanocrystals can be prepared using C18AA derivatives with different carbon chain lengths, as shown in Fig. S7.† The XRD patterns in Fig. S8† reveal that the *d* spacings of the plate-like nanocrystals of C16AA and C14AA are 4.24 and 3.94 nm, respectively. The C16AA and C14AA layers were evaluated to be 3.48 and 3.18 nm, which exceed, but are less than twice the molecular lengths of C16AA and C14AA, respectively (Table S3†). Thus, the plate-like nanocrystals prepared from C16AA and C14AA also have interdigitated structures.

### 3.2. Preparation of perovskite@Pt core–shell nanocrystals

**Fig. 5:**
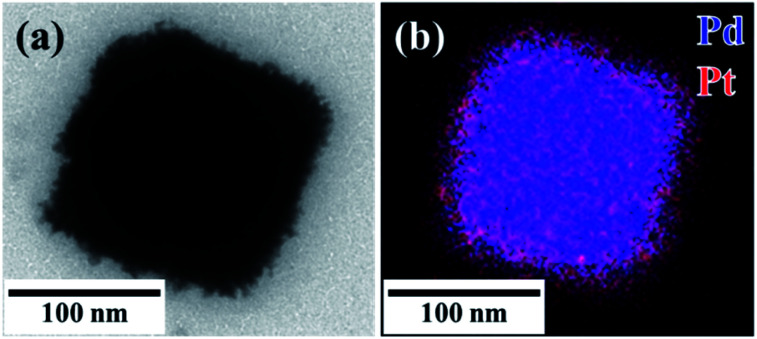
(a) TEM image and (b) scanning TEM map of plate-like perovskite@Pt core–shell nanocrystals. Scale bar is 100 nm.

Seed-mediated growth was used to prepare core–shell (C18AAH_2_)PdCl_4_@Pt nanocrystals from the water-dispersible (C18AAH_2_)PdCl_4_ nanocrystals. The subsequent addition of aqueous K_2_PtCl_4_ (200 mM, 50 μL) and AscA (600 mM, 50 μL) solutions into the as-prepared (C18AAH_2_)PdCl_4_ dispersion resulted in the formation of a black solution. As shown in Fig. 5, S9 and S10,† the surfaces of the plate-like particles became rough due to the deposition of Pt. Fig. 5b also shows that Pt covers the original (C18AAH_2_)PdCl_4_ seed and confirms the formation of (C18AAH_2_)PdCl_4_@Pt core–shell structures.

**Fig. 6:**
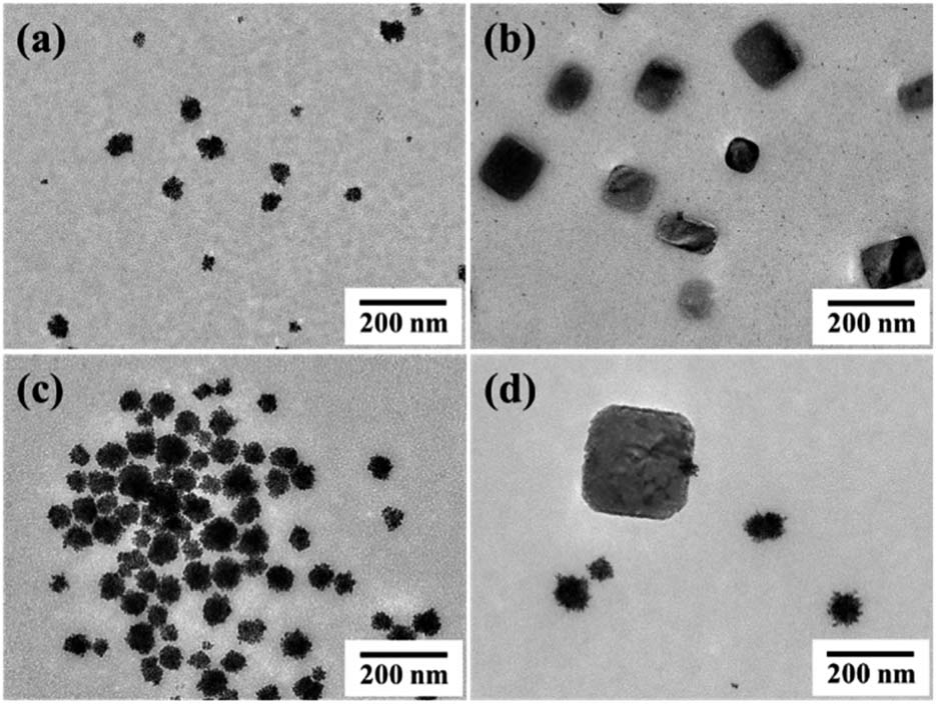
TEM images of plate-like perovskites and Pt nanocrystals obtained when the molar ratio of AscA : K_2_PtCl_4_ was (a and b) 30 : 1, (c) 12 : 1, (d) 6 : 1 under a constant AscA concentration.

In general, the shell structure of core–shell nanoparticles depends on the concentrations of the reducing agent and the metal precursor of the shell as well as the type of reducing agent used.^41,42^ In the present work, these three factors were found to drastically affect the Pt shell structures. For example, under a constant concentration of AscA, the core–shell (C18AAH_2_)PdCl_4_@Pt structure was not obtained at low and high concentrations of K_2_PtCl_4_; at low concentrations of K_2_PtCl_4_ (the molar ratio of AscA : K_2_PtCl_4_ = 6 : 1, 12 : 1 or 30 : 1), Pt nanocrystals and plate-like (C18AAH_2_)PdCl_4_ were produced separately (Fig. 6), while plate-like (C18AAH_2_)PdCl_4_ nanocrystals disappeared and spherical products consisting of Pt cores and Pd shells were obtained at high concentrations of K_2_PtCl_4_ (the molar ratio of AscA : K_2_PtCl_4_ = 1.5 : 1) (Fig. S11†).

When the K_2_PtCl_4_ concentration was kept constant and the AscA concentration varied, (C18AAH_2_)PdCl_4_@Pt core–shell nanocrystals were still obtained at an AscA to K_2_PtCl_4_ molar ratio of 3 (Fig. 5). However, when the concentration ratios of AscA : K_2_PtCl_4_ were 6 : 1, 12 : 1 or 30 : 1, the main products were spherical Pt@Pd core–shell nanocrystals, which were similar to the products obtained at high concentrations of K_2_PtCl_4_ (Fig. S12†). Further, isolated Pt nanocrystals were obtained as minor products when the concentration of AscA was high; the amount of isolated Pt nanocrystals increased with increasing AscA concentration.

Core–shell (C18AAH_2_)PdCl_4_@Pt crystals did not form when the concentration ratio of AscA to K_2_PtCl_4_ was high. This is because more Pt crystals formed in the solution without seed-mediated growth as the amount of reducing agent increased. Hence, the concentration ratio of AscA to K_2_PtCl_4_ is crucial for the formation of core–shell nanocrystals. Furthermore, NaBH_4_, a strong reducing agent, was not effective in producing core–shell structures at all, and the Pt nanoparticles produced in these experiments were not deposited on the core (C18AAH_2_)PdCl_4_ crystals at any molar ratio of NaBH_4_ to K_2_PtCl_4_ (Fig. S13†). Therefore, core–shell structures are not formed when the reduction rate is fast; slow reduction is important for the synthesis of core–shell nanocrystals.

### 3.3. Preparation of perovskite@Au core–shell nanocrystals

**Fig. 7:**
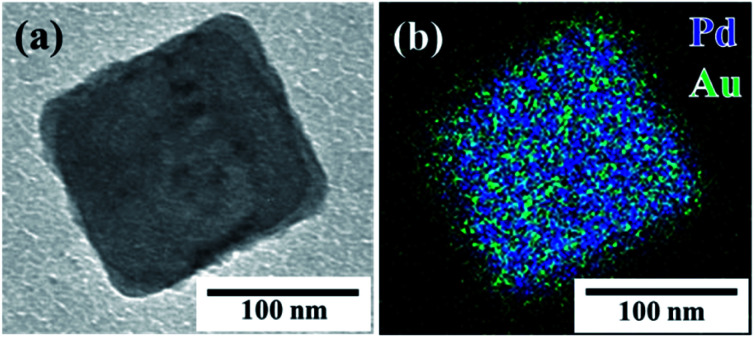
(a) TEM image and (b) scanning TEM map of plate-like perovskite@Au core–shell nanocrystal. Scale bar is 100 nm.

In order to demonstrate that the seed-mediated growth method can also be used to obtain other metal shell structures, we applied the same procedure described above to prepare (C18AAH_2_)PdCl_4_@Au. An AscA to HAuCl_4_ molar ratio of 3 was the best molar ratio for preparing (C18AAH_2_)PdCl_4_@Pt; however, (C18AAH_2_)PdCl_4_ nanoplates completely disappeared and Au@Pd core–shell nanoparticles with an average diameter of 212 nm were produced (Fig. S14†). The disappearance of the plate-like (C18AAH_2_)PdCl_4_ nanocrystals was probably due to a high concentration of HAuCl_4_, which promoted the consumption of (C18AAH_2_)PdCl_4_. In order to find the optimal conditions for preparing (C18AAH_2_)PdCl_4_@Au core–shell nanocrystals, we prepared Au nanocrystals in dispersions of (C18AAH_2_)PdCl_4_ nanoplates at various concentrations of HAuCl_4_ and AscA; (C18AAH_2_)PdCl_4_@Au core–shell nanocrystals were successfully prepared at an AscA to HAuCl_4_ molar ratio of 10. The TEM image in Fig. 7a shows a plate-like nanocrystal covered with a deposited, low-contrast thin layer that consists of Au according to the elemental map in Fig. 7b and the EDS spectrum in Fig. S10.† Thus, the seed-mediated growth method used in this study was effective for the preparation of (C18AAH_2_)PdCl_4_@Au core–shell nanocrystals, even though the optimal conditions were different from those used to synthesise (C18AAH_2_)PdCl_4_@Pt.

## 4. Conclusions

In this study, we demonstrated that water-dispersible, plate-like halide perovskite nanocrystals, which have an average size and thickness of 153 nm and 50 nm, respectively, were prepared by simply mixing C18AA and K_2_PdCl_4_ in water. Moreover, core–shell nanocrystals were synthesised by reducing K_2_PtCl_4_ or HAuCl_4_ with AscA in the presence of the perovskite nanocrystal seeds and the reduction rate was found to be crucial for the formation of these core–shell nanocrystals. These results indicate that C18AA is useful for the preparation of water-dispersible, plate-like perovskites and their core–shell nanocrystals.

## Conflicts of interest

There are no conflicts to declare.

## Supplementary Material

RA-010-D0RA00657B-s001
