# Supplementary material for: Synthesis of water-dispersible, plate-like perovskites and their core–shell nanocrystals
Source: RSC Adv. 2020 Feb 5;10(10):5972–7. doi: 10.1039/d0ra00657b (PMC9049589; doi:10.1039/d0ra00657b)
Supplement: RA-010-D0RA00657B-s001 [file RA-010-D0RA00657B-s001.pdf]

Electronic Supplementary Material (ESI) for RSC Advances.
This journal is © The Royal Society of Chemistry 2020

Electronic Supplementary Information

Synthesis of water-dispersible, plate-like perovskites and their core–shell nanocrystals

Muneharu Minakawa, Yoshiro Imura and Takeshi Kawai*

Department of Industrial Chemistry, Tokyo University of Science
1-3 Kagurazaka, Shinjuku-ku, Tokyo, 162-8601, Japan
kawai@ci.kagu.tus.ac.jp

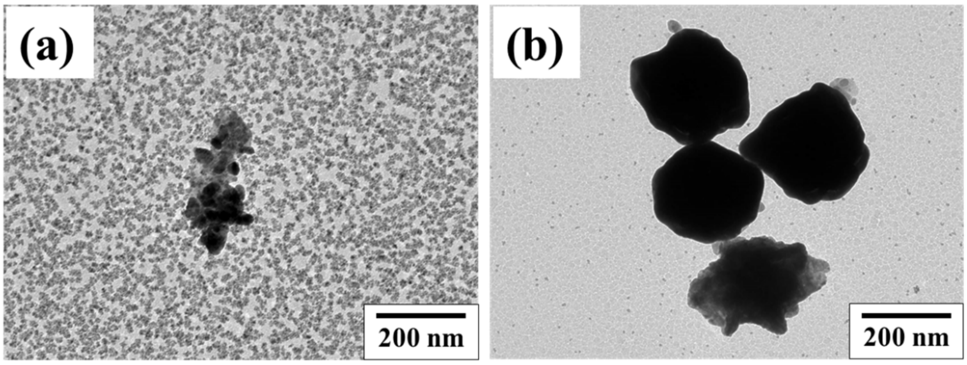

Figure S1. TEM images of nanocrystals after reducing (a) K_2PtCl_4 and (b) $HAuCl_4$ in water containing $(CH_3NH_3)_2PdCl_4$ perovskite.

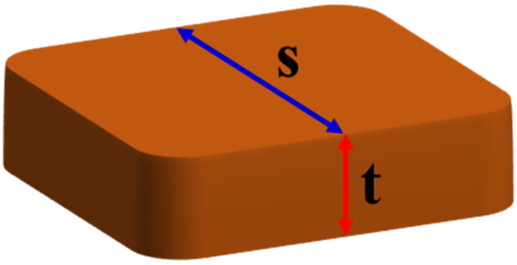

Figure S2. Schematic illustration of a plate-like nanocrystal.

Table S1. Amounts of C, H, N, Pd and Cl in 7.5 mg of $(C18AAH_2)PdCl_4$ determined by CHN elemental analysis and ICP-MS.

	CHN (wt%)	ICP-MS (wt%)	Amount of substance ($\times 10^{-5}$ mol)
C	44.85	-	28.0
H	8.16	-	60.7
N	9.39	-	5.02
Pd	-	14.4	1.01
Cl	-	18.6	3.92

The elemental ratios determined by combined CHN elemental analysis and ICP-MS are in agreement with those determined theoretically.

Table S2. FT-IR band positions of the plate-like nanocrystals. [cm^{-1}]

	CH_2 stretching			
	antisymmetric	symmetric	amide I	amide II
$(C18AAH_2)PdCl_4$	2922	2852	1664	1552
$C18AAH_2Cl_2$	2922	2852	1662	1554
C18AA	2918	2849	1646	1546

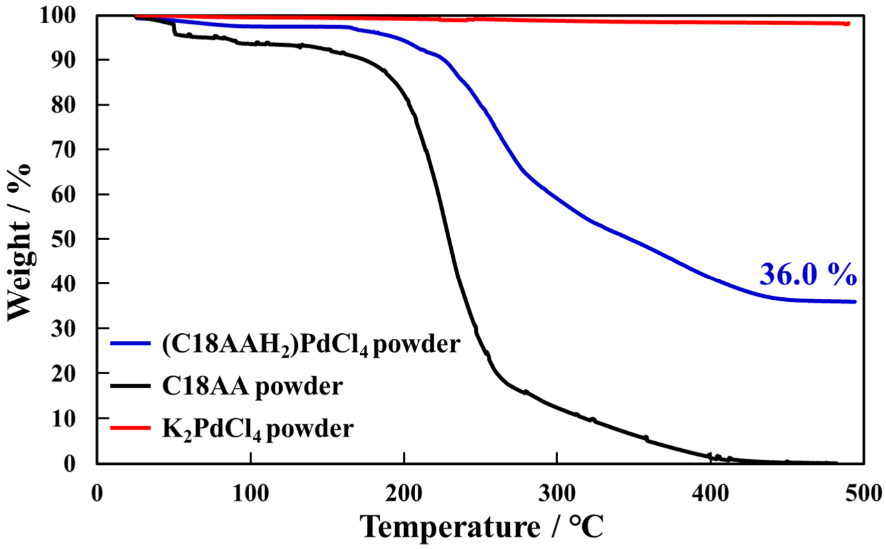

Figure S3. TG thermograms of $(C18AAH_2)PdCl_4$, C18AA and K_2PdCl_4 powders.

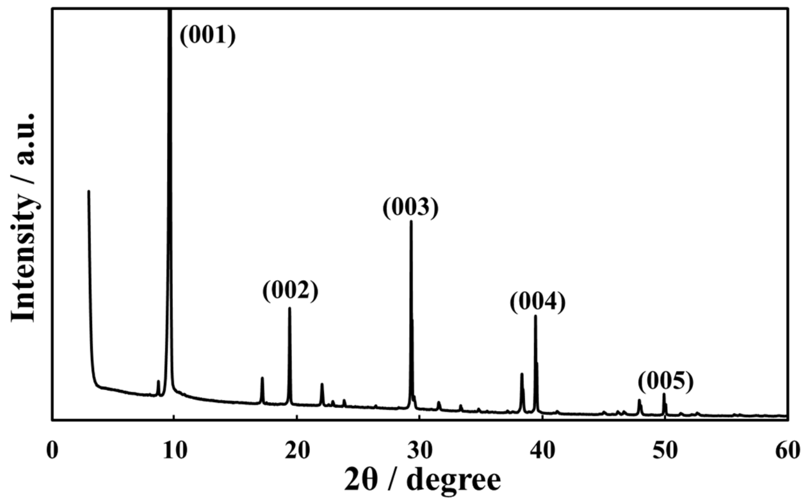

Figure S4. XRD pattern of $(CH_3NH_3)_2PdCl_4$.

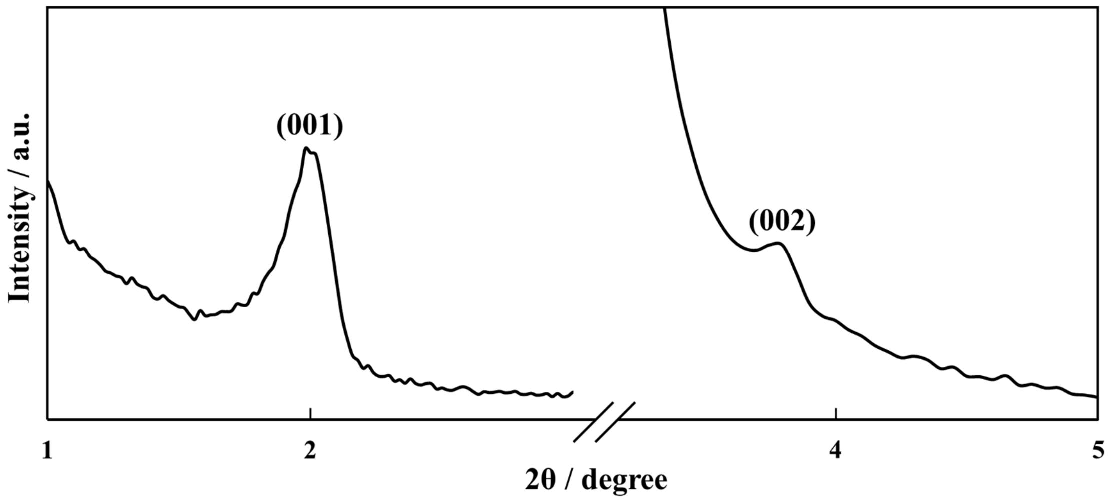

Figure S5. XRD pattern of $(C18AAH_2)PdCl_4$.

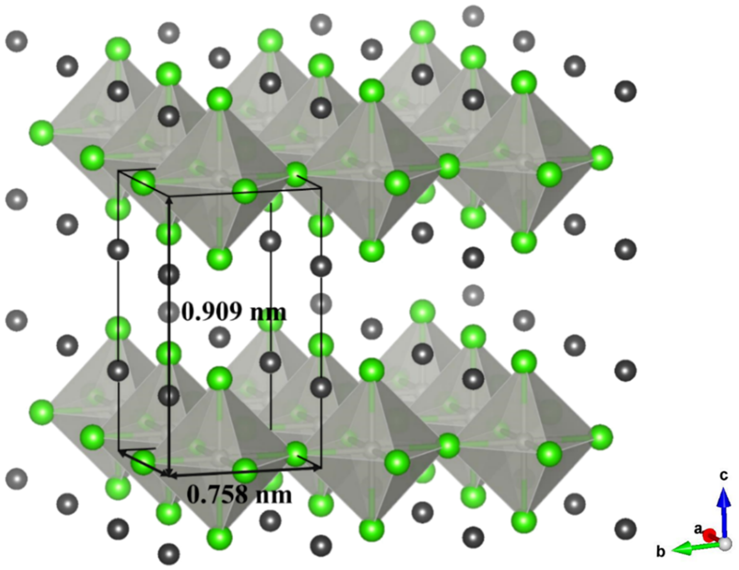

Figure S6. 3D image of the crystal structure of $(CH_3NH_3)_2PdCl_4$.

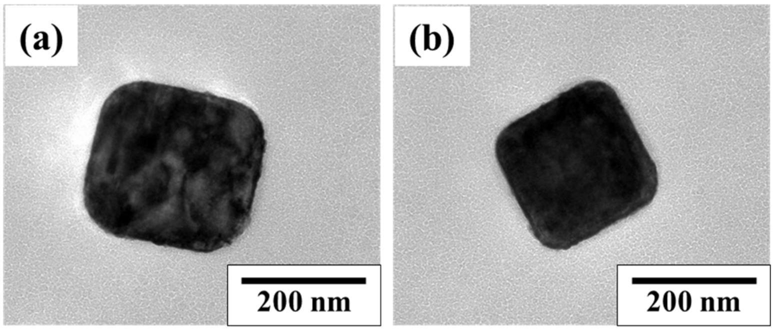

Figure S7. TEM images of plate-like nanocrystals prepared using (a) C16AA and (b) C14AA.

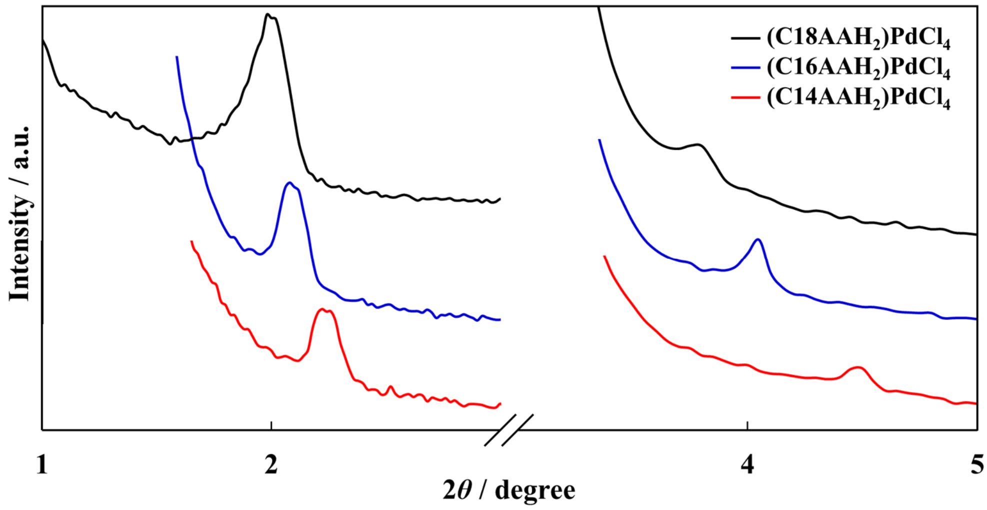

Figure S8. XRD patterns of $(CnAAH_2)PdCl_4$.

Table S3. The *d* spacings of plate-like nanocrystals prepared from C16AA and C14AA.

	θ/deg				
	**first**	**second**	***d* spacing /nm**	**CnAA layer length/nm**	**CnAA molecular length/nm[ref]**
**C18AA**	**1.96**	**3.88**	**4.50**	**3.74**	**2.8**
**C16AA**	**2.08**	**4.05**	**4.24**	**3.48**	**2.6**
**C14AA**	**2.24**	**4.50**	**3.94**	**3.18**	**2.4**

Ref : C. Morita, H. Tanuma, C. Kawai, Y. Ito, Y. Imura, and T. Kawai, *Langmuir*, 2013, **29,** 1669-1675.

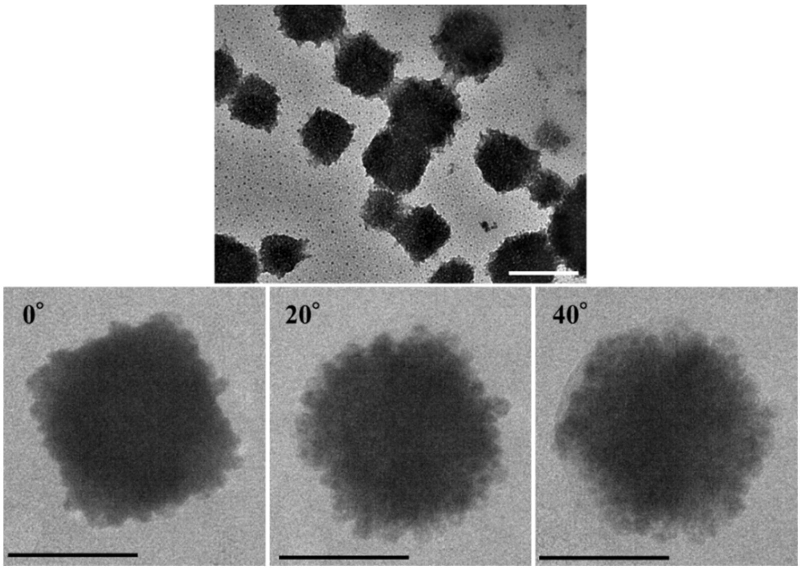

Figure S9. TEM images of $(C18AAH_2)PdCl_4$@Pt core–shell nanocrystals. Scale bar:100 nm.

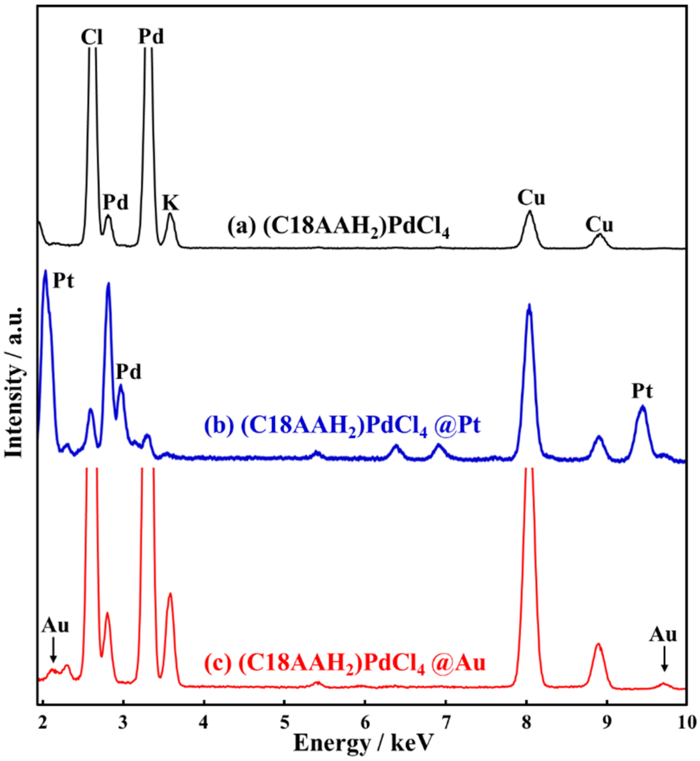

Figure S10. TEM-EDS spectra of (a) $(C18AAH_2)PdCl_4$, (b) $(C18AAH_2)PdCl_4$@Pt and (c) $(C18AAH_2)PdCl_4$@Au core–shell nanocrystals.

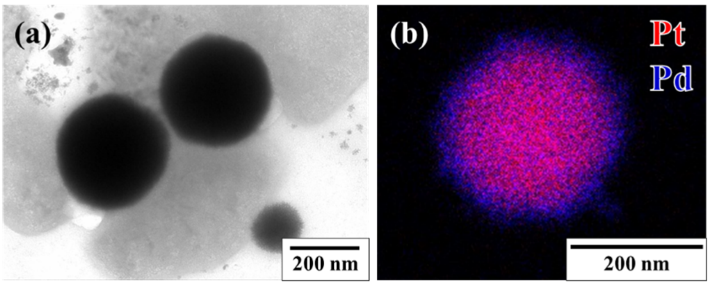

Figure S11. TEM image and scanning TEM elemental map of spherical products obtained when the molar ratio of AscA : K_2PtCl_4 was 1.5:1.

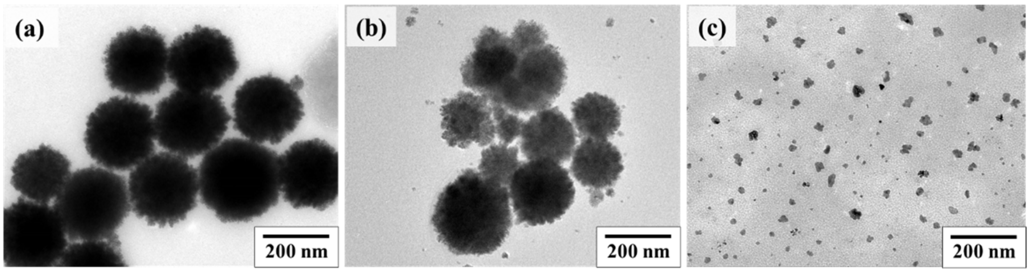

Figure S12. TEM images of the spherical products obtained when the molar ratio of AscA:K_2PtCl_4 was (a) 6:1, (b) 12:1 and (c) 30:1.

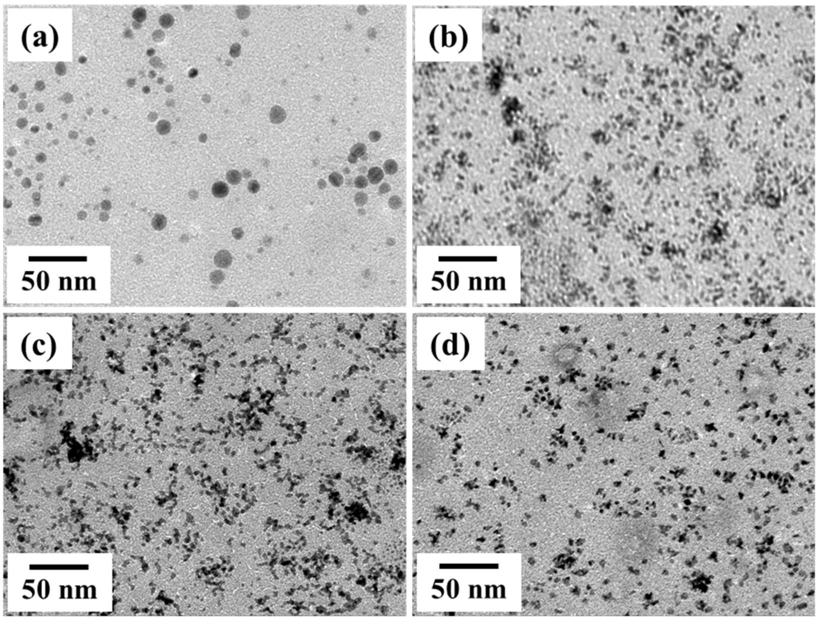

Figure S13. TEM images of Pt nanocrystals obtained when the molar ratio of $NaBH_4$:K_2PtCl_4 was (a) 3:1, (b) 6:1, (c) 12:1 and (d) 30:1.

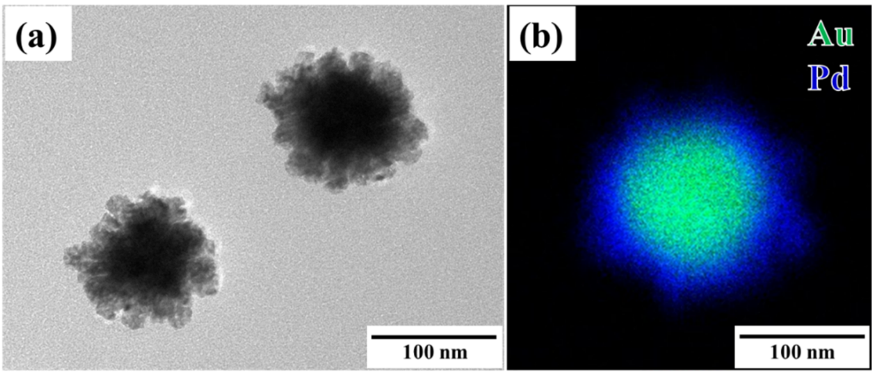

Figure S14. (a) TEM image and (b) scanning TEM map of Au@Pd core–shell nanoparticles obtained when the molar ratio of AscA:$HAuCl_4$ was 3:1.
